# Multimodality echocardiography in structural heart disease: the evolving role of the interventional imager

**DOI:** 10.1093/ehjimp/qyag144

**Published:** 2026-09-03

**Authors:** Nikolaos Ktenopoulos, Konstantina Vlasopoulou, Panayotis Vlachakis, Anastasios Apostolos, Ioannis Skalidis, Paschalis Karakasis, Nikolaos Tsiamis, Theoni Theodoropoulou, Panagiotis Iliakis, Maria Drakopoulou, Andreas Synetos, George Latsios, Konstantina Aggeli, Konstantinos Tsioufis, Konstantinos Toutouzas

**Affiliations:** First Department of Cardiology, National and Kapodistrian University of Athens, Hippokration General Hospital of Athens, Vasilissis Sofias 114, Athens 11527, Greece; Unit of Structural Heart Diseases, First Department of Cardiology, Medical School, National and Kapodistrian University of Athens, Hippocration General Hospital of Athens, Vasilissis Sofias 114, Athens 11527, Greece; First Department of Cardiology, National and Kapodistrian University of Athens, Hippokration General Hospital of Athens, Vasilissis Sofias 114, Athens 11527, Greece; Unit of Structural Heart Diseases, First Department of Cardiology, Medical School, National and Kapodistrian University of Athens, Hippocration General Hospital of Athens, Vasilissis Sofias 114, Athens 11527, Greece; First Department of Cardiology, National and Kapodistrian University of Athens, Hippokration General Hospital of Athens, Vasilissis Sofias 114, Athens 11527, Greece; Unit of Structural Heart Diseases, First Department of Cardiology, Medical School, National and Kapodistrian University of Athens, Hippocration General Hospital of Athens, Vasilissis Sofias 114, Athens 11527, Greece; First Department of Cardiology, National and Kapodistrian University of Athens, Hippokration General Hospital of Athens, Vasilissis Sofias 114, Athens 11527, Greece; Unit of Structural Heart Diseases, First Department of Cardiology, Medical School, National and Kapodistrian University of Athens, Hippocration General Hospital of Athens, Vasilissis Sofias 114, Athens 11527, Greece; Department of Cardiology, Royal Brompton and Harefield Hospitals, Guy’s and St Thomas’ NHS Foundation Trust, London, UK; Department of Cardiology, HFR - Fribourg Cantonal Hospital and University, Fribourg, Switzerland; Second Department of Cardiology, Hippokration General Hospital, Aristotle University of Thessaloniki, Thessaloniki 54642, Greece; First Department of Cardiology, National and Kapodistrian University of Athens, Hippokration General Hospital of Athens, Vasilissis Sofias 114, Athens 11527, Greece; Unit of Structural Heart Diseases, First Department of Cardiology, Medical School, National and Kapodistrian University of Athens, Hippocration General Hospital of Athens, Vasilissis Sofias 114, Athens 11527, Greece; First Department of Cardiology, National and Kapodistrian University of Athens, Hippokration General Hospital of Athens, Vasilissis Sofias 114, Athens 11527, Greece; Unit of Structural Heart Diseases, First Department of Cardiology, Medical School, National and Kapodistrian University of Athens, Hippocration General Hospital of Athens, Vasilissis Sofias 114, Athens 11527, Greece; First Department of Cardiology, National and Kapodistrian University of Athens, Hippokration General Hospital of Athens, Vasilissis Sofias 114, Athens 11527, Greece; First Department of Cardiology, National and Kapodistrian University of Athens, Hippokration General Hospital of Athens, Vasilissis Sofias 114, Athens 11527, Greece; Unit of Structural Heart Diseases, First Department of Cardiology, Medical School, National and Kapodistrian University of Athens, Hippocration General Hospital of Athens, Vasilissis Sofias 114, Athens 11527, Greece; First Department of Cardiology, National and Kapodistrian University of Athens, Hippokration General Hospital of Athens, Vasilissis Sofias 114, Athens 11527, Greece; Unit of Structural Heart Diseases, First Department of Cardiology, Medical School, National and Kapodistrian University of Athens, Hippocration General Hospital of Athens, Vasilissis Sofias 114, Athens 11527, Greece; Faculty of Medicine, European University of Cyprus, Egkomi 2404, Cyprus; First Department of Cardiology, National and Kapodistrian University of Athens, Hippokration General Hospital of Athens, Vasilissis Sofias 114, Athens 11527, Greece; Unit of Structural Heart Diseases, First Department of Cardiology, Medical School, National and Kapodistrian University of Athens, Hippocration General Hospital of Athens, Vasilissis Sofias 114, Athens 11527, Greece; First Department of Cardiology, National and Kapodistrian University of Athens, Hippokration General Hospital of Athens, Vasilissis Sofias 114, Athens 11527, Greece; Unit of Structural Heart Diseases, First Department of Cardiology, Medical School, National and Kapodistrian University of Athens, Hippocration General Hospital of Athens, Vasilissis Sofias 114, Athens 11527, Greece; First Department of Cardiology, National and Kapodistrian University of Athens, Hippokration General Hospital of Athens, Vasilissis Sofias 114, Athens 11527, Greece; Unit of Structural Heart Diseases, First Department of Cardiology, Medical School, National and Kapodistrian University of Athens, Hippocration General Hospital of Athens, Vasilissis Sofias 114, Athens 11527, Greece; First Department of Cardiology, National and Kapodistrian University of Athens, Hippokration General Hospital of Athens, Vasilissis Sofias 114, Athens 11527, Greece; Unit of Structural Heart Diseases, First Department of Cardiology, Medical School, National and Kapodistrian University of Athens, Hippocration General Hospital of Athens, Vasilissis Sofias 114, Athens 11527, Greece

**Keywords:** structural heart disease, transcatheter interventions, interventional echocardiography, three-dimensional echocardiography, intracardiac echocardiography

## Abstract

Over the last decade, transcatheter structural heart interventions (TSHIs) have revolutionized the management of patients with structural heart disease (SHD), particularly those deemed high-risk or inoperable for conventional surgery. This rapid evolution has been propelled by innovations in both device technology and cardiovascular imaging, with echocardiography emerging as a cornerstone for procedural planning, real-time guidance and post-procedural assessment. This review outlines the expanding role of echocardiography across the spectrum of TSHIs, highlighting the importance of multimodality imaging, including three-dimensional transoesophageal echocardiography (3D TEE), intracardiac echocardiography (ICE) and fusion imaging techniques, for comprehensive structural evaluation and intraprocedural navigation. As the field continues to evolve, the demand for imaging specialists with procedural expertise is growing. Challenges remain in optimizing workflow, minimizing radiation exposure and enhancing image-guided precision. This review aims to outline the development of interventional imaging and promote best practices for procedural success and patient safety.

## Graphical Abstract

**Graphical Abstract:**
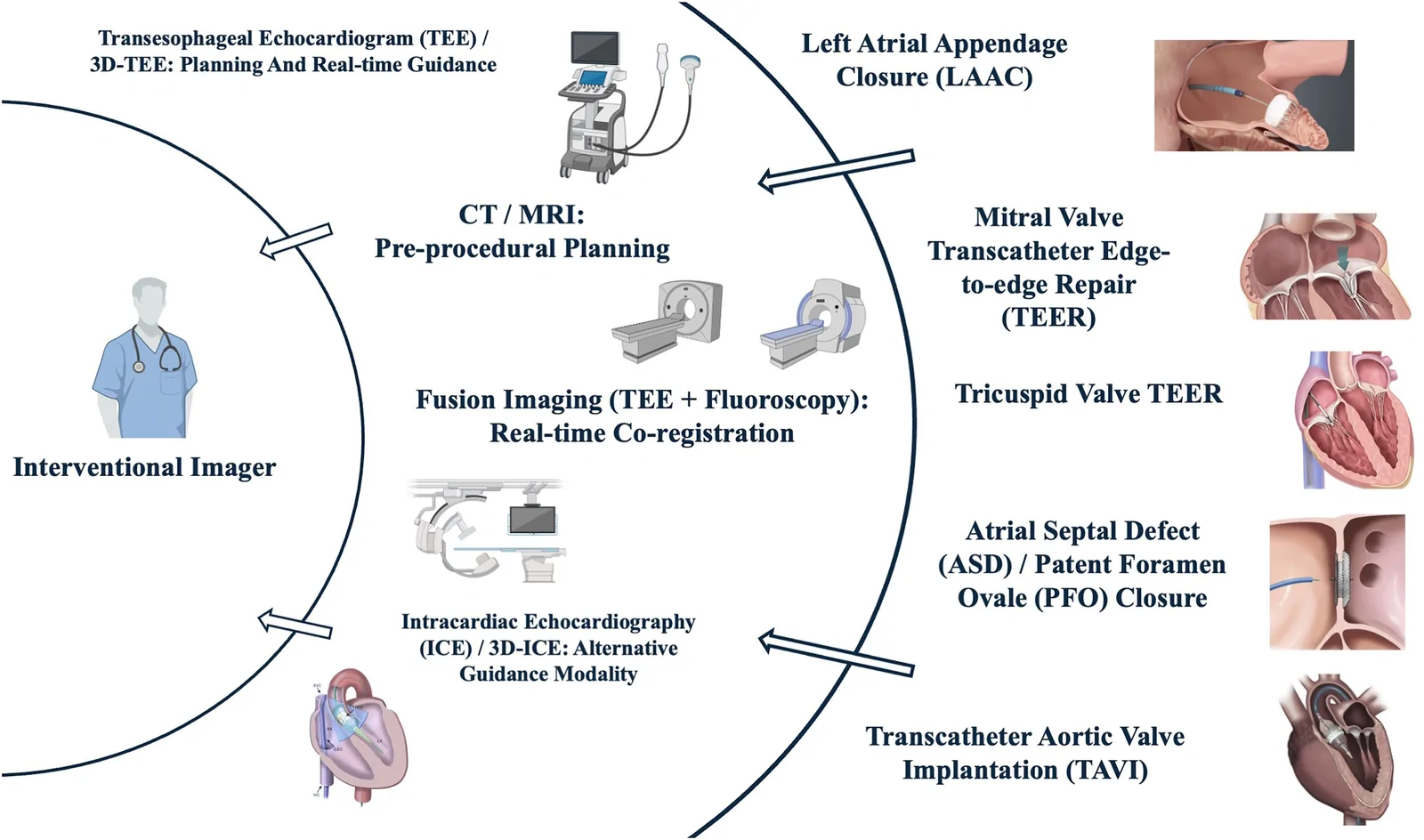

## Introduction

Structural heart disease (SHD), encompassing a wide array of congenital and acquired valvular and septal abnormalities, has been revolutionized by the rapid development of transcatheter structural heart interventions (TSHIs).^1,2^ This field has witnessed a global surge, exemplified by a nearly 10-fold increase in annual TAVI procedures over the last decade, with projections indicating continued growth.^3–7^ These procedures have become a clinical cornerstone for high-risk patients formerly deemed inoperable.

Because of the complexity of the cardiac anatomy, procedural success and patient safety during TSHIs are entirely dependent on advanced imaging guidance. Echocardiography has emerged as the pivotal tool for pre-procedural planning, real-time intraprocedural navigation and post-intervention assessment. The transition from two-dimensional to high-resolution 3D/4D datasets has been a ‘game-changer’, providing the temporal dimension necessary for dynamic cardiac motion assessment. Contemporary practice increasingly utilizes a multimodality approach, integrating computed tomography (CT), cardiac magnetic resonance (CMR) and intracardiac echocardiography (ICE) to support minimalist, sedation-sparing procedures. This evolution has birthed the ‘interventional imager’, a specialized expert who functions as an integral member of the multidisciplinary heart team, optimizing workflows and reducing cumulative radiation exposure while ensuring procedural precision.^1,8^ The aim of this review is to highlight the integral role of the interventional imager and echocardiography during TSHIs and promote best practices for procedural success and patient safety.

## Imaging tools for structural interventions: echocardiography

The landscape of TSHIs has been fundamentally reshaped by the transition from traditional two-dimensional (2D) imaging to advanced three-dimensional (3D) and four-dimensional (4D) echocardiographic datasets. While 3D echocardiography provides the high-resolution spatial visualization required to understand complex cardiac anatomy, the addition of the temporal dimension in 4D imaging enables the real-time dynamic assessment of cardiac motion and function.

Transoesophageal Echocardiography (TEE) remains the primary modality for intraprocedural guidance, particularly for atrioventricular interventions. Modern TEE platforms now utilize 3D multiplanar reconstruction (3D-MPR), which permits for direct 2D observation of the anatomy in many simultaneous views, effectively minimizing errors of parallax. Furthermore, photorealistic imaging techniques, such as transillumination, employ virtual light sources to enhance the sense of depth and space. These innovations allow interventional imagers to detect subtle anatomical pathologies and refine the visualization of regurgitant jets or shunts by superimposing colour Doppler onto high-fidelity anatomic data.^9–14^

Parallel to advancements in TEE, ICE has emerged as a pivotal tool for facilitating a ‘minimalist approach’. By utilizing steerable ultrasound catheters introduced via femoral venous access, ICE provides high-resolution imaging from within the cardiac chambers. The shift toward 3D and 4D ICE has been particularly transformative, offering near-TEE-quality imaging while allowing procedures to be performed under local anaesthesia or conscious sedation. This avoids the inherent risks of general anaesthesia and oesophageal injury associated with TEE, which is especially beneficial for elderly or frail populations. ICE is now frequently utilized for transseptal puncture (TSP) guidance, left atrial appendage occlusion (LAAO) and increasingly for complex mitral and tricuspid repairs. These innovations make 3D-ICE a promising alternative to TEE for simpler procedures and valuable adjunct during more complex interventions. In particular, 3D-ICE is expected to play an increasingly important role in transcatheter mitral and tricuspid valve interventions, where it can complement TEE by improving visualization of leaflet anatomy, device–tissue interaction and leaflet grasping when TEE image quality is suboptimal.^15–18^

Nevertheless, ICE presents some challenges, including probe instability in a hyperdynamic cardiac environment and limited imaging depth. Furthermore, its use may be hindered by vascular complications, risk of arrythmias and financial barriers such as inadequate reimbursement policies. Further advancements in ICE technology are necessary for it to be adopted as a routine, stand-alone imaging tool for guiding TSHIs. Despite the absence of universally accepted procedural protocols, several institutions have developed their own guidelines.^17–20^

Another significant technological frontier is echo-fluoroscopy fusion imaging. This hybrid technique involves the co-registration of TEE or ICE datasets with live fluoroscopic projections, aligning them in both time and space. By overlaying soft-tissue anatomical models onto the fluoroscopic screen, fusion imaging enhances procedural navigation and device positioning. This integration not only improves procedural precision but also serves as a critical safety feature by reducing cumulative radiation exposure for both patients and staff and minimizing the volume of contrast medium required.^21–24^

Finally, while echocardiography is the cornerstone of real-time guidance, it must be viewed within a multimodality framework. The integration of pre-procedural CT and CMR is now essential for comprehensive planning, such as annular sizing for transcatheter valve replacement or identifying myocardial fibrosis. Collectively, these advanced techniques have necessitated the formalization of the interventional imager as a distinct subspecialty within the heart team, requiring specialized training in real-time 3D interpretation and multidimensional navigation.^11–18,20–24^  *Table 1* summarizes the TEE views, probe positions and manoeuvres used by the interventional imager during the periprocedural evaluation of TSHIs.^1,9–12,25,26^

**Table 1:** Summary of periprocedural transoesophageal echocardiographic views, probe position and manoeuvres for transcatheter structural heart interventions

*Procedure*	*Key echocardiographic views*	*Probe position*	*Primary imaging objectives*
*TSP*	4C, short-axis, RV inflow-outflow, bicaval, 3D LA views	Mid-oesophageal	Fossa ovalis localization; needle tenting confirmation; 3D-MPR/fusion imaging guidance
*PFO/ASD Closure*	4C, short-axis, bicaval, 3D en face of IAS	Mid-oesophageal	3D/balloon defect sizing; rim assessment; PFO bubble study; device alignment and deployment
*Mitral Valve TEER (e.g. MitraClip)*	4C, bicommissural, 2C, long-axis, LVOT, 3D en face MV	Mid-oesophageal to TG	Device steering and leaflet grasping; clip orientation and deployment; residual MR assessment
*Mitral VIV*	4C, bicommisural, long-axis, LVOT, 3D MV annular	Mid-oesophageal and deep TG	Valve positioning and alignment, LVOT obstruction assessment; PVL evaluation
*PTMC*	4C, long-axis, short-axis at MV level, 3D en face MV	Mid-to-deep oesophageal and TG	Balloon guidance; MVA measurement; post-procedurural MR evaluation
*Tricuspid Valve TEER*	Bicaval, 4C, 5C, RV inflow-outflow, 3D en face TV, TG long-axis, short-axis	Mid-to-deep oesophageal and TG	Challenging imaging, often requires ICE; Leaflet visualization and grasping; jet localization; residual TR assessment
*Tricuspid Annuloplasty Device (e.g. K-Clip)*	Bicaval, 4C, 5C, RV inflow-outflow, 3D en face TV, TG long-axis, short-axis	Mid-to-deep oesophageal and TG	Annular sizing; device–leaflet interaction assessment; anchor deployment guidance
*TAVR*	Long-axis, LVOT, short-axis at AV level, TG long-axis, deep TG 5C	Mid-oesophageal, *trans*-gastric, deep TG	Annulus sizing; valve deployment assessment; PVL and complication detection; TTE procedure guidance preferred
*PVL Closure*	3D en face of prosthetic valve, colour Doppler, short-axis	Prosthetic valve specific	3D/3D-MPR for PVL location and sizing; device selection; residual PVL assessment
*LAAO*	0°, 45°, 90°, 135° multi-plane views, 3D LAA views	Mid-oesophageal	3D-MPR for precise LAA anatomy, landing zone, and implantation depth evaluation; residual leak assessment

Abbreviations: 2C, 2-chamber; 3D, three-Dimensional; 4C, 4-chamber; 5C, 5-chamber; ASD, Atrial Septal Defect; AV, Aortic Valve; IAS, Interatrial Septum; ICE, Intracardiac Echocardiography; LA, Left Atrium; LAA, Left Atrial Appendage; LAAO, Left Atrial Appendage Occlusion; LVOT, Left Ventricular Outflow Tract; MPR, Multiplanar Reconstruction; MR, Mitral Regurgitation; MV, Mitral Valve; MVA, Mitral Valve Area; PFO, Patent Foramen Ovale; PTMC, Percutaneous Mitral Commissurotomy; PVL, Paravalvular Leak; RV, Right Ventricle; TAVR, Transcatheter Aortic Valve Replacement; TEE, Transoesophageal Echocardiography; TEER, Transcatheter Edge-To-Edge Repair; TG, Trans-Gastric; TSP, Transseptal Puncture; TV, Tricuspid Valve; VIV, Valve-In-Valve.

## The interventional echocardiographer: a new standalone subspecialty

The rapid expansion of TSHIs has necessitated the formalization of the interventional imager as a standalone subspecialty. Unlike conventional diagnostic imaging, interventional echocardiography (IE) requires the real-time interpretation of non-conventional views, with 3D reconstruction being pivotal for procedural guidance.

Competency in this field is distinct from general echocardiography and requires a mastery of spatial orientation to guide catheters and devices safely. Professional societies in USA and Europe emphasize that core competencies must include not only technical proficiency but also high-level multidisciplinary communication within the Heart Team. For specific tools like ICE, a steep learning curve exists. Guidelines recommend a minimum of 10 supervised procedures to achieve basic competence in catheter manipulation, though significantly higher volumes are required for the independent guidance of complex repairs.

Current training models are shifting toward a hybrid approach, merging the interventionalist’s manual dexterity with the imaging specialist’s interpretive expertise. Despite the growing demand, many regions still lack a structured core curriculum, highlighting an urgent need for standardized educational endeavours and regional networking to ensure procedural success and patient safety.^1,8,27,28^

## Radiation safety considerations for the interventional imager

The successful execution of TSHIs relies on a robust multidisciplinary Heart Team approach. This team, comprising interventionalists, cardiac surgeons and specialized interventional imagers, facilitates shared decision-making by balancing clinical evidence with patient preferences. This collaboration is particularly vital in complex scenarios where high-level evidence may be sparse, ensuring that procedural benefits outweigh risks.

Safety considerations are paramount, especially regarding radiation exposure. Imagers are at high-risk due to their proximity to the X-ray source, necessitating the use of protective lead shielding, distancing and monitored dosing. Crucially, real-time echocardiography (TEE and ICE) enhances safety by reducing cumulative radiation burden and fluoroscopy time.

Furthermore, meticulous continuous monitoring enables the immediate detection of complications, such as pericardial effusions, thrombus formation, or device malposition. The shift toward minimalist procedures via ICE further improves safety by avoiding the risks of general anaesthesia and oesophageal injury associated with TEE, which is beneficial for elderly or frail populations. This integrated approach optimizes outcomes while prioritizing patient and staff safety.^1,8^

## The heart team approach to transcatheter mitral valve management

The management of mitral valve (MV) disease has undergone a paradigm shift, moving from a primarily surgical focus to a complex, multidisciplinary framework necessitated by the explosion of TSHIs. As nearly 50% of patients with symptomatic mitral regurgitation (MR) are historically denied surgical operations especially the more elder, with more comorbidities, or reduced ventricular function, and the Heart Team approach has become the clinical gold standard for navigating this expanded therapeutic landscape.^29–31^

### Treatment of mitral regurgitation

Transcatheter Edge-to-Edge Repair (TEER) of the MV, based on the surgical Alfieri stitch method and most frequently performed with the MitraClip or the Pascal device, has emerged as a less invasive alternative for patients with significant MR who are considered unsuitable for surgery.^32,33^ Despite some initial barriers, the approach have steadily gained momentum over the past decade.

For optimal patient selection, a comprehensive anatomical and functional assessment is required. Traditional echocardiographic parameters, including leaflet coaptation length, coaptation depth, flail height and flail width, remain important for evaluating procedural feasibility, as they influence the likelihood of achieving durable leaflet capture and adequate MR reduction. Historically, successful TEER has been associated with a coapting surface length ≥2 mm and a coaptation depth ≤11 mm in functional MR and with a flail height <10 mm and flail width <15 mm in degenerative MR.^9^ However, with contemporary TEER systems incorporating wider clip widths and longer arm lengths, these measurements should be considered guidance parameters rather than absolute selection criteria. In current practice, patient suitability is determined through multidisciplinary Heart Team discussion integrating clinical and imaging findings. Particular attention is paid to baseline *trans*-mitral gradients and mitral valve area (MVA), as a *trans*-mitral gradient <5 mmHg (at a heart rate of 60–80 beats/min) and an MVA ≥4.0 cm^2^ are desirable to minimize the risk of post-procedural mitral stenosis.^34^

**Figure 1:**
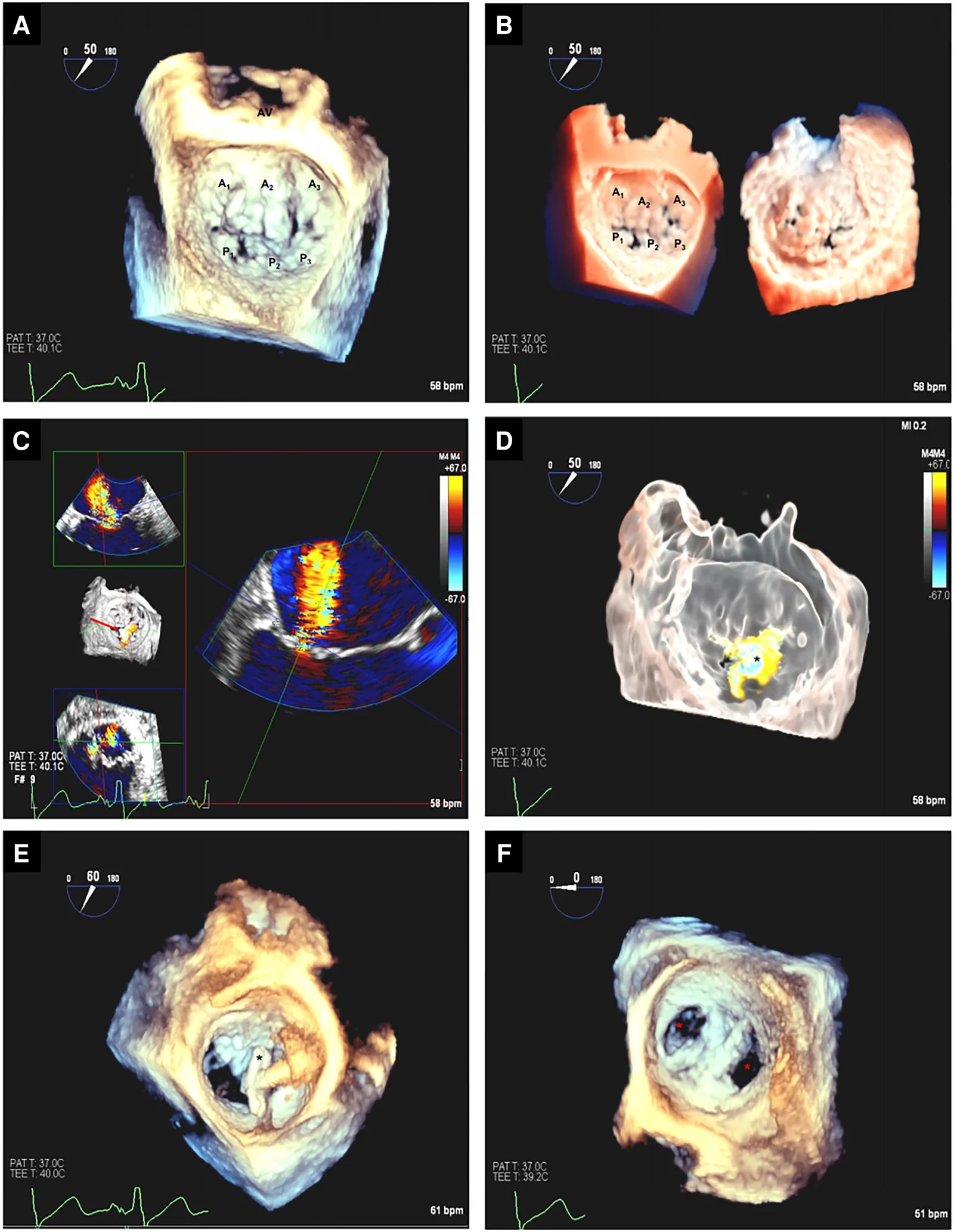
3D TEE image of the mitral valve in ‘zoom mode’ showing anatomy from the atrial perspective, with the aortic valve at the 12 o’clock position. The large anterior leaflet (A1, A2, A3) and shorter posterior leaflet (P1, P2, P3) are shown from lateral to medial orientation. Coaptation gaps appear as dark spaces along the coaptation line and between the P1 and P2 scallops (*A*). The same view in 3D transillumination imaging (TI) dual mode provides atrial (left) and ventricular (right) perspectives, enhancing leaflet visualization with ‘light’ mode and demonstrating mitral valve tenting (*B*). A pre-procedural 3D cine full-volume colour Doppler loop with multiple image reconstructions reveals severe secondary mitral regurgitation (red arrow) with restriction of the posterior mitral leaflet (*C*). The broad regurgitant jet is visualized across the coaptation margin (asterisk) in the 3D ‘glass’ mode, highlighting leaflet tenting and the regurgitant jet origin (*D*). During MitraClip deployment, 3D TEE ensures correct clip orientation (asterisk) perpendicular to the coaptation line (*E*). Two clips were placed—one lateral to the other—to address the broad jet. Post-procedural 3D TTE shows a double-orifice mitral valve (asterisks) with two parallel, well-deployed clips (*F*).^1^ AV, aortic valve.^35^

Key echocardiographic views for procedural planning and guidance include the mid-oesophageal four-chamber, bicommissural, two-chamber and long-axis views (*Table 1*). 3D TEE provides high quality illustration of the mitral segments, is superior to 2D TEE for the assessment of degenerative MR and facilitates communication with the interventional cardiologist. Accurate interpretation of leaflet morphology requires careful optimization of image settings, as excessive gain may obscure leaflet edges and overestimate tissue thickness, whereas insufficient gain may create apparent leaflet defects or underrepresent leaflet coaptation. Correlation with MPR and colour Doppler is therefore essential. The use of biplane imaging and 3D-MPR modalities can further increase technical success and shorten procedural times. While the TSP technique used is similar to that for other left-sided interventions, the puncture location is tailored to the target pathology and device trajectory. For MV TEER, a posterior and superior puncture site is generally preferred because it provides sufficient left atrial working space, facilitates coaxial alignment of the delivery catheter with the line of leaflet coaptation and improves device manoeuvrability during steering and grasping. Suboptimal puncture location may limit catheter mobility, compromise leaflet access and increase procedural complexity. 3D-MPR and fusion echocardiography-ﬂuoroscopy imaging can facilitate precise TSP site selection and optimize procedural efficiency. Key procedural steps such as device steering, leaflet grasping and clip deployment require meticulous real-time multiplanar echocardiographic guidance to confirm success.^1,10–12^  *Figure 1* illustrates comprehensive 3D TEE and transillumination imaging of the mitral valve during MitraClip implantation, demonstrating leaflet anatomy, regurgitant jet localization and post-procedural double-orifice formation.

### Transcatheter mitral valve-in-valve procedures

With bioprosthetic MV replacements becoming increasingly common, structural degeneration of these prostheses is also rising.^36^ For patients deemed high-risk for redo surgery, transcatheter mitral valve-in-valve (VIV) implantation offers a viable solution. This approach, often utilizing balloon-expandable valves. Contemporary practice favours a transfemoral transseptal approach whenever feasible, owing to its less invasive nature and favourable procedural and recovery profile compared with transapical access.

**Figure 2:**
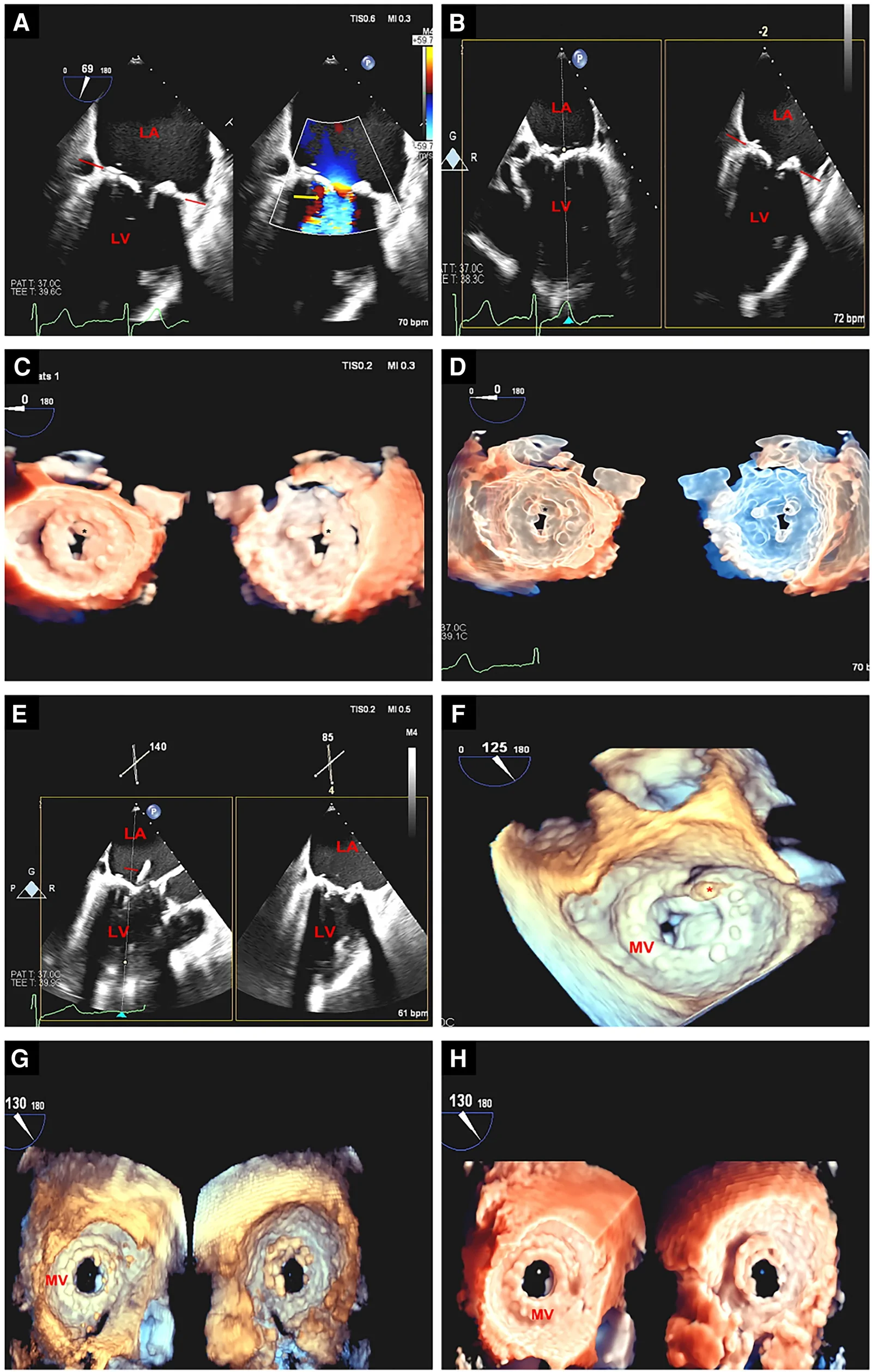
2D TEE bicommissural views with and without colour Doppler demonstrate severely thickened and restricted bioprosthetic mitral valve leaflets (red arrows) with diastolic turbulent flow (yellow arrow) (*A*). The 2D TEE biplane image further confirms marked leaflet restriction in diastole, consistent with degenerative bioprosthetic stenosis (*B*). Real-time 3D TEE images using 3D transillumination imaging (TI) TrueView (*C*) and glass-view (*D*) projections from atrial (left) and ventricular (right) perspectives highlight restricted leaflet motion (asterisk), confirming prosthetic degeneration. Intraprocedural 2D TEE imaging shows the guidewire traversing the stenotic mitral prosthesis (red arrow) before valve delivery (*E*), while the 3D atrial ‘en face’ view confirms correct guidewire positioning (asterisk) between the anterior and lateral prosthetic leaflets (*F*). Post-deployment standard (*G*) and TrueView (*H*) 3D TEE images from both perspectives display the implanted 29 mm Edwards Sapien valve-in-valve prosthesis.^1^ LA, left atrium; LV, left ventricle; MV, mitral valve.^35^

Pre-procedural multidetector computed tomography (MDCT) is critical to measure the mitral annulus and predict the neo-left ventricular outflow tract (LVOT) area to prevent obstruction, as it remains one of the most feared and potentially life-threatening complications of TMVR. When using the transseptal route, TSP is classically TEE guided and the puncture location is typically posterior and mid-septum. After successful puncture, the transcatheter heart valve (THV) is delivered and deployed under the combined guidance of fluoroscopy and TEE (*Table 1*). Post-deployment, echocardiographic monitoring is essential to detect complications like LVOT obstruction, which might necessitate further interventions such as surgical bailout or septal ablation.^37–39^  *Figure 2* illustrates the stepwise echocardiographic assessment and guidance of a degenerative bioprosthetic mitral valve treated with a VIV procedure.

### Treatment of mitral stenosis

In the setting of rheumatic MS, the Heart Team evaluates suitability for Percutaneous Transvenous Mitral Commissurotomy (PTMC), which is the standard of care for patients with favourable anatomy. If anatomical or clinical contraindications for PTMC exist (e.g. LAA thrombus or unfavourable Wilkin’s score), mitral surgery is recommended.

For degenerative MS associated with extensive mitral annular calcification (MAC), the options are often limited and carry higher risk. The Heart Team may consider Transcatheter Mitral Valve Implantation (TMVI) in selected symptomatic high-risk patients, but this requires meticulous pre-procedural planning with Cardiac CT to assess the risk of LVOT obstruction (*Table 1*). While 3D TTE may slightly overestimate MVA compared with 3D TEE, the latter provides superior image quality and detailed visualization of commissural anatomy.

During PTMC, TEE-guided TSP is a critical procedural step. After septal crossing, the Inoue balloon catheter is advanced across the stenotic MV under TEE and fluoroscopic guidance. Colour Doppler imaging helps assess the emergence of any contemporary Mv regurgitation that may occur following the inflation of the balloon. Additional echocardiographic techniques, such as 2D TEE in short-axis view or 3D ‘en-face’ visualization, allow for detailed evaluation of commissural splitting and precise planimetric measurement of the mitral valve area. If the post-dilation results are suboptimal, additional inflations may be performed.

Procedural success is generally defined by an increase in MVA to >1.5 cm^2^ (often measured intra-procedurally using TEE 3D-MPR), together with a significant reduction in *trans*-mitral gradients, improvement in pulmonary pressures and the absence of severe post-procedural MR. TEE remains critical in detecting adverse events like pericardial effusion, tamponade, or intracardiac thrombus creation during and following the procedure.^12,40–44^

## Multimodality navigation of the interatrial septum

**Figure 3:**
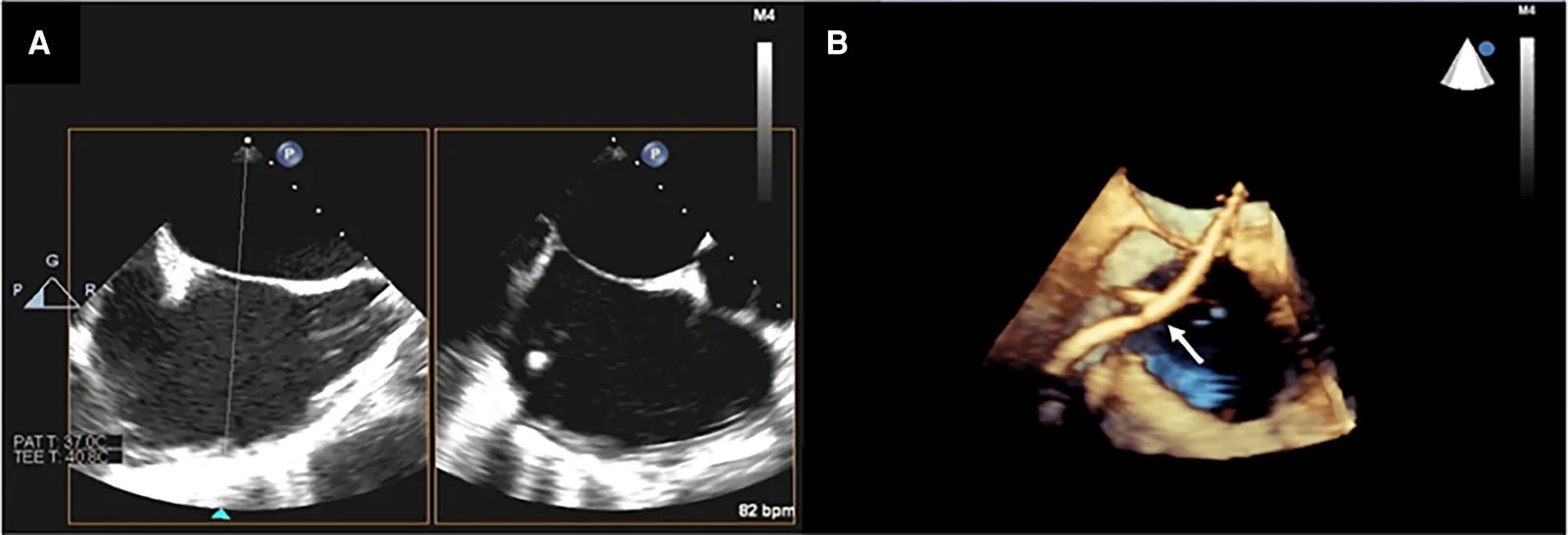
After acquiring the 2D TEE bicaval view (*A*), multiplanar reconstruction encompassing the entire IAS and adjacent cardiac structures is performed (*B*). This reconstruction enhances visualization of the spatial relationship between the IAS and surrounding anatomy, facilitating precise tracking of the catheter.^35^

The interatrial septum (IAS) serves as the fundamental ‘gateway’ for the majority of left-sided TSHIs, including MV TEER, LAAO and paravalvular leak (PVL) closure. The fossa ovalis (FO), which constitutes almost one fifth of the IAS, remains the sole structure that can be penetrated with caution without the fear of entering the extracardiac space. Because direct visualization of this anatomy is impossible during transcatheter procedures, the interventional imager’s role in providing real-time guidance is critical for procedural success and complication avoidance^12,45–49^ (*Figure 3*).

### Imaging guidance for transseptal puncture

Precise localization of the puncture site is mandatory to ensure an optimal trajectory for the specific device being delivered. TEE remains a cornerstone, utilizing the mid-esophageal bicaval view (80°–100°) for superior-inferior orientation and the short-axis view (30°–45°) for anterior-posterior orientation. The transition to 3D/4D-TEE and multiplanar reconstruction (MPR) has been a ‘game changer,’ allowing the imager to visualize the ‘tenting’ of the septum in multiple planes simultaneously, which minimizes errors of parallax (*Tables 1* and *2*).^1,25,50,51^

**Table 2:** Procedure-specific transseptal puncture sites

Procedure	Ideal TSP site
Mitral Valve TEER	Posterior and mid to superior for central MR jets; Higher TSP heights for more medial MR jets; Lower TSP sites for lateral MR jets
Mitral VIV	Posterior and mid-septum
PTMC	Posterior and mid-septum
PVL Closure	Case-dependent
LAAO	Inferior and mid to posterior

Abbreviations: LAAO, Left Atrial Appendage Occlusion; MR, Mitral Regurgitation; PTMC, Percutaneous Mitral Commissurotomy; PVL, Paravalvular Leak; TEER, Transcatheter Edge-To-Edge Repair; TSP, Transseptal Puncture; VIV, Valve-In-Valve.

The specific puncture site must be tailored to the intervention: a posteroinferior puncture is generally preferred for LAAO to facilitate coaxial alignment, whereas a posterosuperior puncture (∼4 cm above the mitral annulus) is required for MV TEER. ICE has emerged as a robust alternative for TSP guidance, providing high-resolution ‘Home,’ ‘Bicaval’ and ‘Aortic Short-Axis’ views from within the right atrium. ICE is particularly advantageous for identifying anatomical variants, such as a thick or aneurysmal septum, and allows for real-time needle monitoring.^17,18,25,51^

### Atrial septal defect (ASD) assessment and closure

Secundum ASDs are the most common form of interatrial communication (75–80%) and are the primary targets for transcatheter closure. Pre-procedural evaluation requires a comprehensive assessment of right heart size, function, and shunt fraction (Qp/Qs). While TTE serves as an initial screening tool, TEE is essential for detailed anatomical characterization due to its proximity to the IAS.

The success of percutaneous closure depends on the meticulously measured size of the defect and the adequacy of the surrounding anatomical rims. Current guidelines suggest evaluating six specific rims at 15° increments. A rim is considered deficient if it measures <5 mm. Deficiencies in rims other than the superior-anterior (aortic) rim often necessitate surgical rather than transcatheter management. 3D/4D-TEE provides incremental value by displaying the ‘en-face’ view of the defect, allowing the imager to appreciate its dynamic changes in size and shape throughout the cardiac cycle, a phenomenon known as the ‘dynamic ASD’. Intraprocedurally, sizing balloons are used to determine the ‘stop-flow’ diameter, ensuring the selection of an appropriately sized occluder.

### Patent foramen ovale (PFO) and the minimalist approach

**Figure 4:**
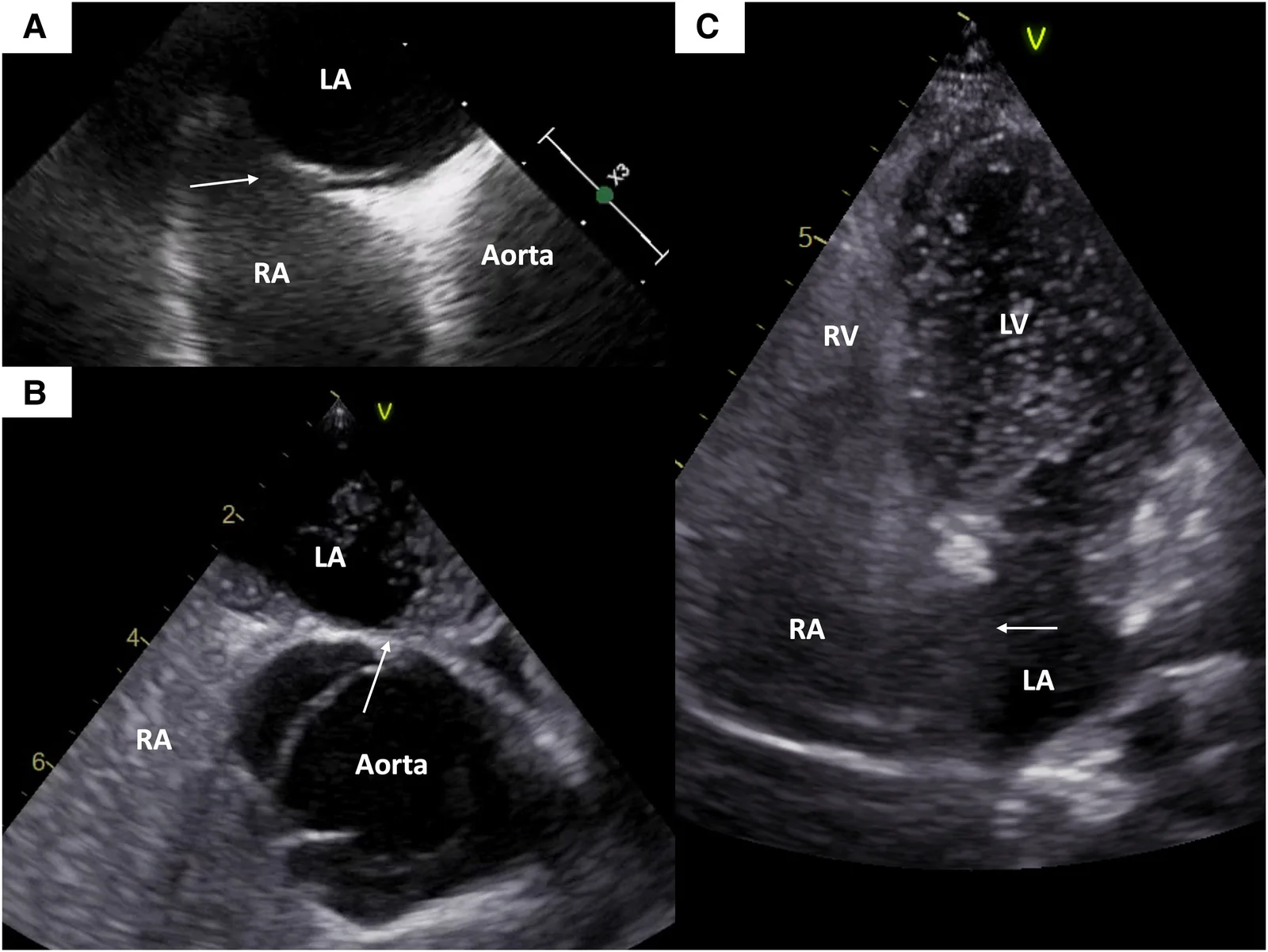
Echocardiographic assessment of patent foramen ovale (PFO) using bubble contrast test. (*A*) Two-dimensional transoesophageal echocardiography (TEE) image demonstrating transient leftward bowing (‘septal bounce’) of the IAS toward the left atrium (LA, arrow) following release of the Valsalva manoeuvre, indicating a pressure gradient favouring right-to-left shunting. (*B*) Contrast-enhanced TEE showing microbubble passage from the right atrium (RA) through a patent foramen ovale into the LA (arrow), confirming the presence of an interatrial communication. (*C*) Transthoracic echocardiography (TTE) images obtained during contrast study demonstrating right-to-left shunt upon Valsalva release, with leftward deviation of the atrial septum (arrow). LA, left atrium; RA, right atrium; LV, left ventricle; RV, right ventricle.

PFOs are functional tunnels between the septum primum and secundum, affecting ∼25% of the population. They are implicated in cryptogenic strokes, particularly in patients under 65 years of age. Diagnosis relies on contrast-enhanced TTE or TEE bubble studies to isolate the shunt at the atrial level and quantify its degree. Careful optimization of gain settings is important during septal imaging, as insufficient gain may create dropout artefacts that mimic or exaggerate septal discontinuity, whereas excessive gain can overestimate defect dimensions and obscure tissue borders. Confirmation of defect size and morphology using multiple imaging planes, 3D imaging and colour Doppler is therefore recommended before device selection (*Figure 4*).

During the intervention, TEE assists in determining the correct sizing of the occlusion device and monitors its positioning and deployment. Simultaneously, long segments of the catheters and wires and their relationship with surrounding structures are displayed. Ensuring that the device discs are aligned parallel to the IAS is vital. A ‘push-and-pull’ manoeuvre (or so-called ‘wiggle manoeuvre’) is often used to confirm the device stability prior to release. Final TEE evaluation involves confirming complete septal closure, absence of residual shunt and exclusion of complications such as pericardial effusion, thrombus formation, device erosion or migration.^25,52–54^

The field is rapidly shifting toward a ‘minimalist approach’ for PFO and ASD closures, catalyzed by the integration of 4D ICE. ICE allows these procedures to be performed under local anaesthesia or conscious sedation, avoiding the inherent risks of general anaesthesia and oesophageal injury associated with TEE. This approach is especially beneficial for elderly or frail populations, leading to shorter recovery times and improved workflow efficiency.^25,52–54^

### Safety and complication monitoring

Meticulous imaging guidance is the primary defense against procedural complications. Real-time TEE and ICE facilitate the immediate detection of rare but serious events, such as cardiac tamponade (incidence 0.2–1.5%), aortic puncture, or device embolization. Furthermore, the use of advanced echocardiography reduces cumulative radiation exposure and fluoroscopy time, protecting both the patient and the catheterization laboratory staff. Post-procedural assessment with colour Doppler and agitated saline is essential to adjudicate the success of the closure and exclude significant residual shunts.^1,12,17,18,25,45–54^

### Role of ICE

ICE is a robust intraprocedural alternative for guiding PFO and ASD closures, providing high-resolution visualization from within the right atrium. By utilizing steerable catheters, operators obtain critical septal and long-axis views to evaluate anatomical rims, measure ‘stop-flow’ diameters during balloon sizing and monitor device deployment in real time. A primary advantage over TEE is the facilitation of a ‘minimalist approach’. Despite these benefits, limitations include a narrower field of view, higher costs for single-use catheters, the need for additional venous access, and a steep learning curve requiring specialized training. Consequently, ICE serves as an essential complementary tool, particularly in experienced programs when precise rim definition is mandatory.^18,20,25,52^

## The heart team strategy for selecting the mode of intervention for aortic valve disease

Transcatheter aortic valve implantation (TAVI) has revolutionized aortic stenosis management, expanding from high-risk patients to younger, lower-risk populations. Success is predicated on a multimodality imaging framework. Cardiac CT is the gold standard for pre-procedural planning, providing precise measurements of the aortic annulus, coronary ostia height and peripheral access.

For patients with CT contraindications, 3D/4D TEE offers a high-resolution alternative for annular sizing. Intraprocedurally, TEE provides real-time guidance for device positioning, mitigating risks like coronary obstruction or the ‘Mt. Fuji’ sign indicating potential annular rupture. Post-deployment, the interventional imager utilizes Doppler echocardiography to adjudicate success, specifically screening for PVL and transvalvular gradients. As TAVI evolves toward complex bicuspid and VIV interventions, this integrated imaging approach remains essential for optimizing prognostic outcomes and patient safety (*Table 1*).^1,12,29–33,55–58^

## Imaging the ‘forgotten valve’: advancing tricuspid valve care with 3d/4d TEE and ICE

The tricuspid valve (TV), historically labelled the ‘forgotten valve’, has emerged as a critical frontier in interventional cardiology due to the recognition that significant tricuspid regurgitation (TR) is associated with increased mortality and poor clinical outcomes. TV TSHIs now offer less invasive options for high-risk patients, but their success is entirely predicated on a sophisticated multimodality imaging framework. The complex, saddle-shaped, and elliptical geometry of the TV annulus, combined with high anatomical variability, where only 57% of patients possess the classic three-leaflet configuration, requires the high-resolution spatial and temporal visualization provided by 3D and 4D echocardiography. Although traditionally described as comprising three leaflets (anterior, posterior and septal), contemporary imaging studies have demonstrated considerable anatomical variability, with accessory leaflet scallops and four-leaflet configurations frequently encountered. Accurate characterization of leaflet morphology and commissural anatomy has become increasingly important in the era of TV TSHIs, as these features may influence device selection, procedural planning and intraprocedural guidance.^59–65^

### Pre-procedural assessment and grading

A comprehensive evaluation begins with TTE as the first-line screening tool to determine TR aetiology and its impact on right-sided chambers. However, 3D TEE is mandatory to visualize all three leaflets simultaneously and identify the specific mechanism of TR. To refine the adjudication of procedural success, contemporary guidelines have expanded the grading scale beyond ‘severe’ to include ‘massive’ and ‘torrential’ TR.^66^

Accurate grading of TR severity is fundamental for patient selection and assessment of procedural outcomes. Beyond the traditional classification of mild, moderate and severe TR, contemporary recommendations recognize the additional categories of massive and torrential TR to better characterize advanced disease frequently encountered in patients referred for TSHI.^67^ This expanded grading scheme has important implications for procedural planning and outcome assessment, as patients with massive or torrential TR often present with larger coaptation gaps, more advanced right heart remodelling and lower procedural success rates. Furthermore, meaningful clinical benefit may be achieved despite residual TR when a substantial reduction in TR severity is obtained.^68–70^

Evidence shows that favourable anatomical predictors for successful TEER include a coaptation depth no more than 10 mm, coaptation gap <7.2 mm (ideally <4 mm), leaflet lengths more than 10 mm and central or anterior-septal jet direction, as these characteristics facilitate leaflet grasping and durable reduction of TR. Conversely, severe tethering or enlarged regurgitant orifice may predict suboptimal results. Small or retracted septal leaflets often preclude clip-based approaches.^26,71^

While TEE remains the gold standard for procedural guidance, a multimodality imaging strategy is essential for patient selection and procedural planning in TV TSHIs. MDCT allows high-resolution visualization of annular geometry, spatial relationships to the right coronary artery (RCA) and access routes. CMR provides precise quantification of RV volumes, function and regurgitant fraction and is particularly valuable for assessing RV remodelling. In patients with contraindications to CMR, including those with non-CMR-compatible pacemaker or defibrillator leads, 3D TTE represents an important alternative for evaluating RV size, function and geometry. The integration of these modalities ensures comprehensive anatomical and functional assessment of the TV and right heart structures.^66^

**Figure 5:**
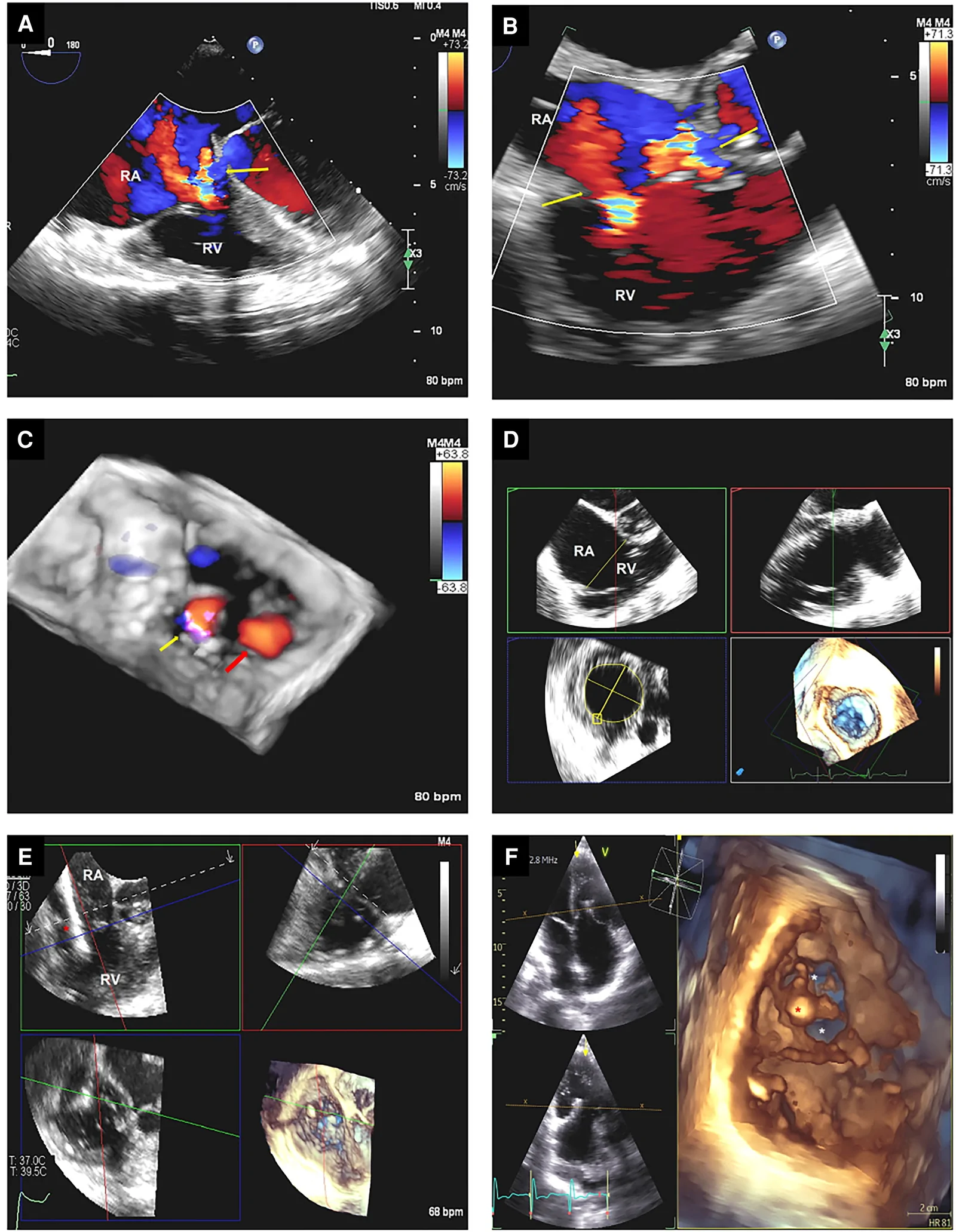
Preoperative 2D TEE images at the gastroesophageal junction of the tricuspid valve at 0° (*A*) and 70° (*B*) with colour Doppler demonstrate significant annular dilatation and severe tricuspid regurgitation with two distinct jets (yellow arrows). Real-time 3D TEE imaging with colour flow confirms the presence of two regurgitant jets (*C*): one between the posterior and septal leaflets (red arrow) and another between the anterior and septal leaflets (yellow arrow). A 3D TEE multiplanar reconstruction prior to clip placement delineates annular dimensions (yellow line) and valve area (yellow circle) (*D*). Intraprocedural multiplanar reconstruction identifies the delivery system (asterisk) crossing into the right ventricle between the anterior and septal leaflets (*E*). Post-procedural 3D TEE images from the right ventricular perspective demonstrate a double-orifice tricuspid valve (white asterisks) with optimal clip deployment (red asterisk) (*F*).^1^ RA, right atrium; RV, right ventricle.^35^

Furthermore, the Heart Team increasingly relies on CT and CMR for holistic evaluation. CMR remains the gold standard for quantifying right ventricular (RV) volumes and ejection fraction, while CT is indispensable for annular sizing and mapping the proximity of the RCA to avoid iatrogenic injury during annuloplasty.^26,71–73^  *Figure 5* demonstrates the multimodality TEE guidance of transcatheter tricuspid valve repair, highlighting annular assessment, jet localization and post-procedural confirmation of clip positioning.^74–76^

### Intraprocedural guidance with 3d/4d TEE

3D/4D TEE is the primary modality for guiding atrioventricular interventions like TEER. Successful leaflet grasping depends on identifying the ‘en-face’ perspective of the TV, standardized with the aortic valve at 10 o’clock and the inferior vena cava at 7 o’clock. The use of MPR and 3D-MPR is a ‘game changer,’ allowing the imager to visualize the delivery system and the valve plane simultaneously in multiple views, thereby minimizing errors of parallax.

Photorealistic imaging and transillumination enhance depth perception, helping the interventional imager detect subtle anatomical pathologies and refine device positioning. Specifically for TEER, imaging must confirm anatomical suitability, as a coaptation depth <10 mm and a coaptation gap <7 mm are independent predictors of success. Post-deployment, TEE is used to adjudicate success by measuring transvalvular gradients and screening for residual TR or complications like pericardial effusion.

### The rise of 4D intracardiac echocardiography

The landscape of TV interventions is rapidly shifting toward a ‘minimalist approach’ enabled by 4D ICE. By utilizing steerable phased-array ultrasound catheters introduced via femoral access, ICE provides high-resolution imaging directly from within the RA. This facilitates procedures under local anaesthesia or conscious sedation, avoiding the risks of general anaesthesia and oesophageal injury associated with TEE, which is particularly beneficial for frail or elderly populations.

Next-generation 3D and 4D ICE systems now provide real-time volumetric imaging and near-TEE-quality visualization of the septal, anterior and posterior leaflets. From the RA ‘home view’, the probe can be oriented anteriorly to monitor leaflet grasping and device orientation while minimizing shadowing from adjacent structures. While TEE currently maintains superior spatial resolution, the autonomy ICE gives the interventionalist and the streamlined workflow it facilitates make it an increasingly central tool for right-sided interventions.

The transition of the TV from ‘forgotten’ to a central clinical focus has been propelled by the integration of 3D/4D TEE, ICE and multimodality frameworks. The emergence of the specialized interventional imager within the Heart Team ensures that complex anatomical variations are navigated with precision, cumulative radiation exposure is reduced and patient safety is prioritized. As 4D ICE and fusion imaging technologies continue to mature, the precision and safety of TV TSHIs will continue to advance, further improving quality of life and promoting reverse RV remodelling for these complex patients.^26,59–61,71–73,77^

## Pulmonary valve interventions

Pulmonary valve (PV) TSHIs are most often performed in congenital heart disease, typically for residual stenosis, regurgitation, or mixed dysfunction after right ventricular outflow tract (RVOT) repair. Transcatheter PV replacement (TPVR) offers a less invasive alternative to repeat surgery for failing RV, pulmonary artery conduits or degenerated bioprostheses, including in patients with prohibitive surgical risk such as pregnancy. TTE and TEE assess gradients and prosthesis-patient mismatch (PPM), but PV imaging may be limited by its anterior position. CMR is the reference standard for RV volumes, function, regurgitant fraction and RVOT anatomy. MDCT complements procedural planning by providing detailed assessment of RVOT morphology, conduit dimensions and the spatial relationship between the RVOT and the coronary arteries. Particular attention should be paid to the risk of left main or left anterior descending coronary artery compression, which represents a potentially catastrophic complication of TPVR and should be excluded by CT and/or invasive balloon interrogation with simultaneous coronary angiography. 3D printing can support complex-case simulation further.^26,73,78–80^

## Stroke prophylaxis beyond anticoagulation: the interventional imager’s role in LAAO

LAAO has become a vital stroke prophylaxis strategy for patients with atrial fibrillation who are unsuitable for long-term oral anticoagulation. Procedural success is fundamentally dependent on the interventional imager, whose role begins with meticulous pre-procedural planning. Cardiac CT and 3D TEE are integrated to evaluate complex appendage morphologies, such as windsock, cauliflower, or chicken-wing, and to define the ‘landing zone’ for device selection.^10,81–84^

**Figure 6:**
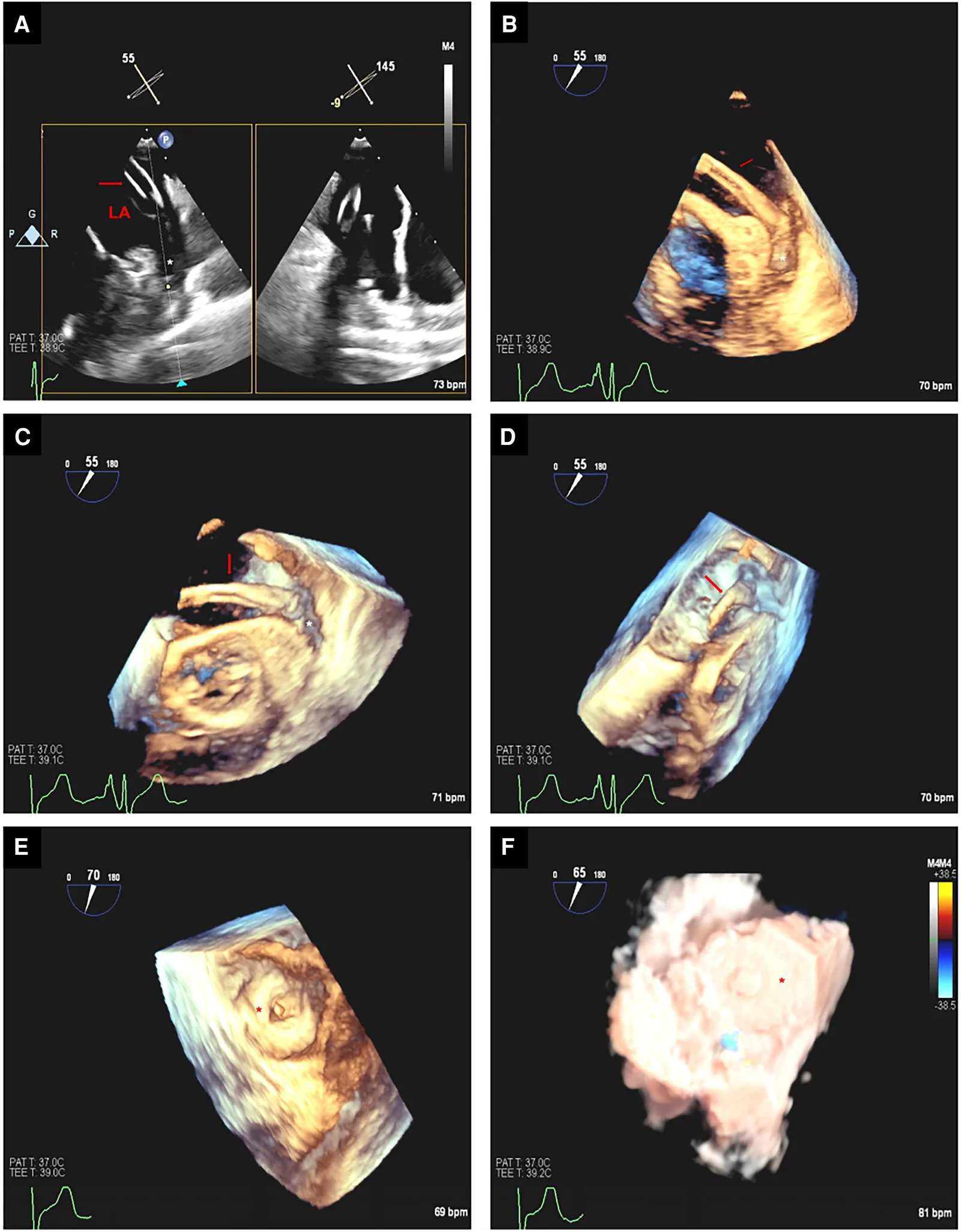
Mid-oesophageal TEE images depict a 2D *x*-plane view at 55° and 145°, guiding the catheter (red arrow) into the left atrial appendage (asterisk) (*A*). Real-time 3D-TEE multiplanar reconstruction from these views further facilitates catheter guidance into the left atrial appendage (*B–D*). Postprocedure, 3D-TEE imaging shows the occlusion device fully expanded within the left atrial appendage (asterisk) (*E*), with TrueView imaging confirming the absence of significant residual flow between the left atrium and left atrial appendage (*F*).^1^ LA, left atrium.^35^

Intraprocedural guidance traditionally relies on 3D TEE, utilizing 3D MPR to obtain unbiased measurements of the LAA segments, including the ostium, neck and lobe. The ostial dimension is typically measured almost two centimetres from the apex of the left upper pulmonary vein limbus. Transillumination and photorealistic rendering enhance depth perception, allowing the imager to precisely guide the TSP and pigtail catheter placement while identifying subtle anatomical variations. Final imaging confirms device stability and rules out complications such as thrombus formation, pericardial effusion, or *trans*-mitral and pulmonary vein ﬂow obstruction.^1,10,12,84–88^  *Figure 6* shows 2D and 3D TEE guidance of a LAAO procedure.

The landscape of LAAO is rapidly shifting toward a minimalist approach facilitated by 4D ICE. Furthermore, echo-fluoroscopy fusion imaging (co-registration) aligns these datasets in time and space, improving procedural navigation and reducing cumulative radiation exposure.^17,84,88,89^ Post-deployment, the imager must adjudicate success by assessing for residual shunts using colour Doppler and monitoring for immediate complications such as device malposition, thrombus formation, or pericardial effusion. This multimodality framework ensures that LAAO is both safe and effective, providing long-term protection against thromboembolic events.

## Advanced imaging for paravalvular leak closure

PVL closure is a highly echo-dependent procedure targeting regurgitant jets through defects between a prosthetic valve and the native annulus. While many defects are small, significant leaks in 1–5% of patients lead to congestive heart failure or haemolytic anaemia, necessitating an interventional approach. Advanced 3D/4D TEE is the gold standard for every procedural stage, from initial defect characterization to final adjudication.^90–92^

**Figure 7:**
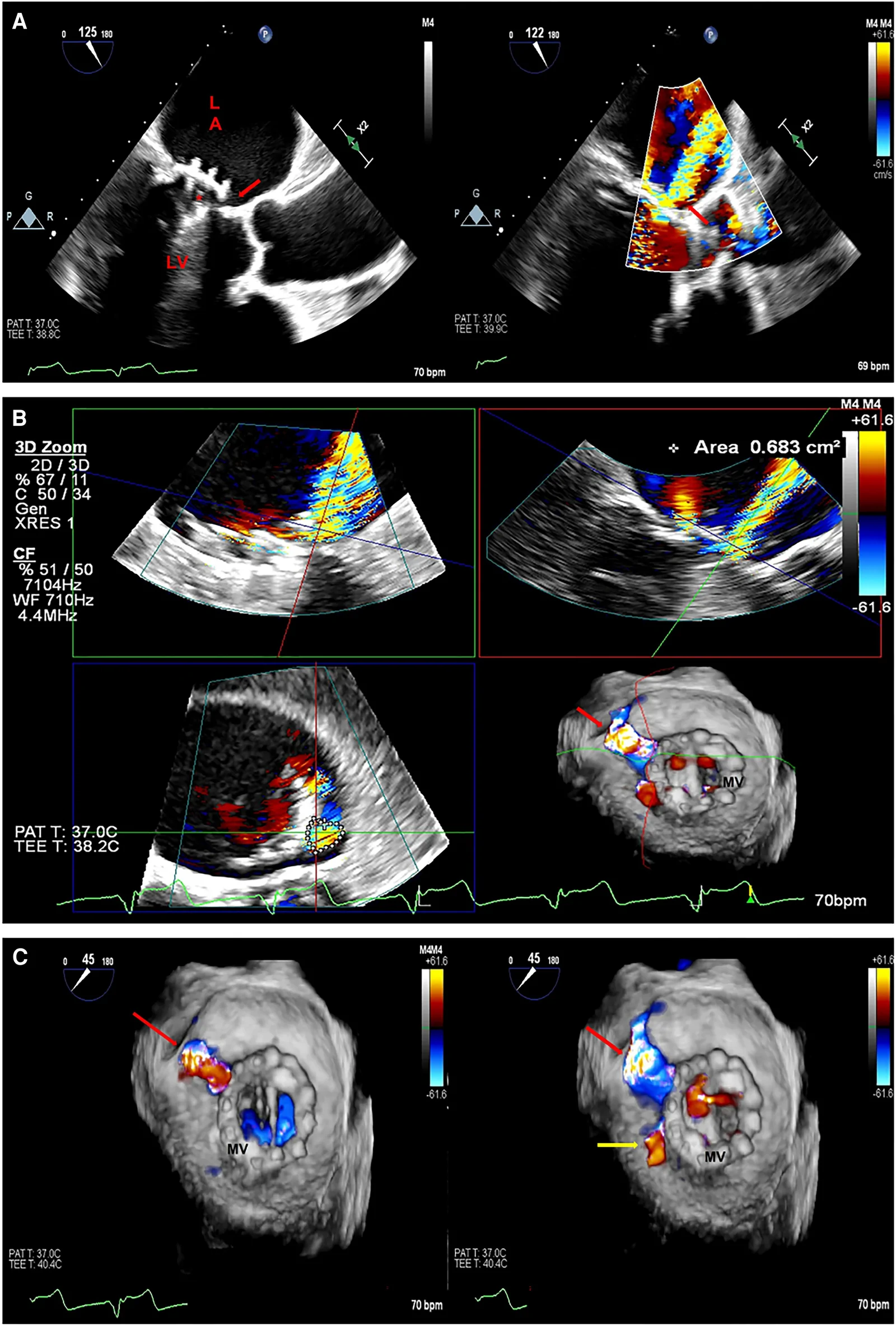
2D TEE long-axis view with and without colour Doppler shows a mechanical mitral prosthesis (asterisk) with anterolateral paravalvular leak (red arrow) (*A*). Three-dimensional multiplanar reconstruction confirms the anterolateral periprosthetic regurgitation (red arrow) (*B*). Real-time 3D-TEE colour Doppler from the atrial perspective demonstrates the anterolateral regurgitation adjacent to the prosthesis at eleven o’clock (red arrow) and a smaller posterolateral defect at nine o’clock (yellow arrow) (*C*).^1^ LA, left atrium; LV, left ventricle; MV, mitral valve.^35^

**Figure 8:**
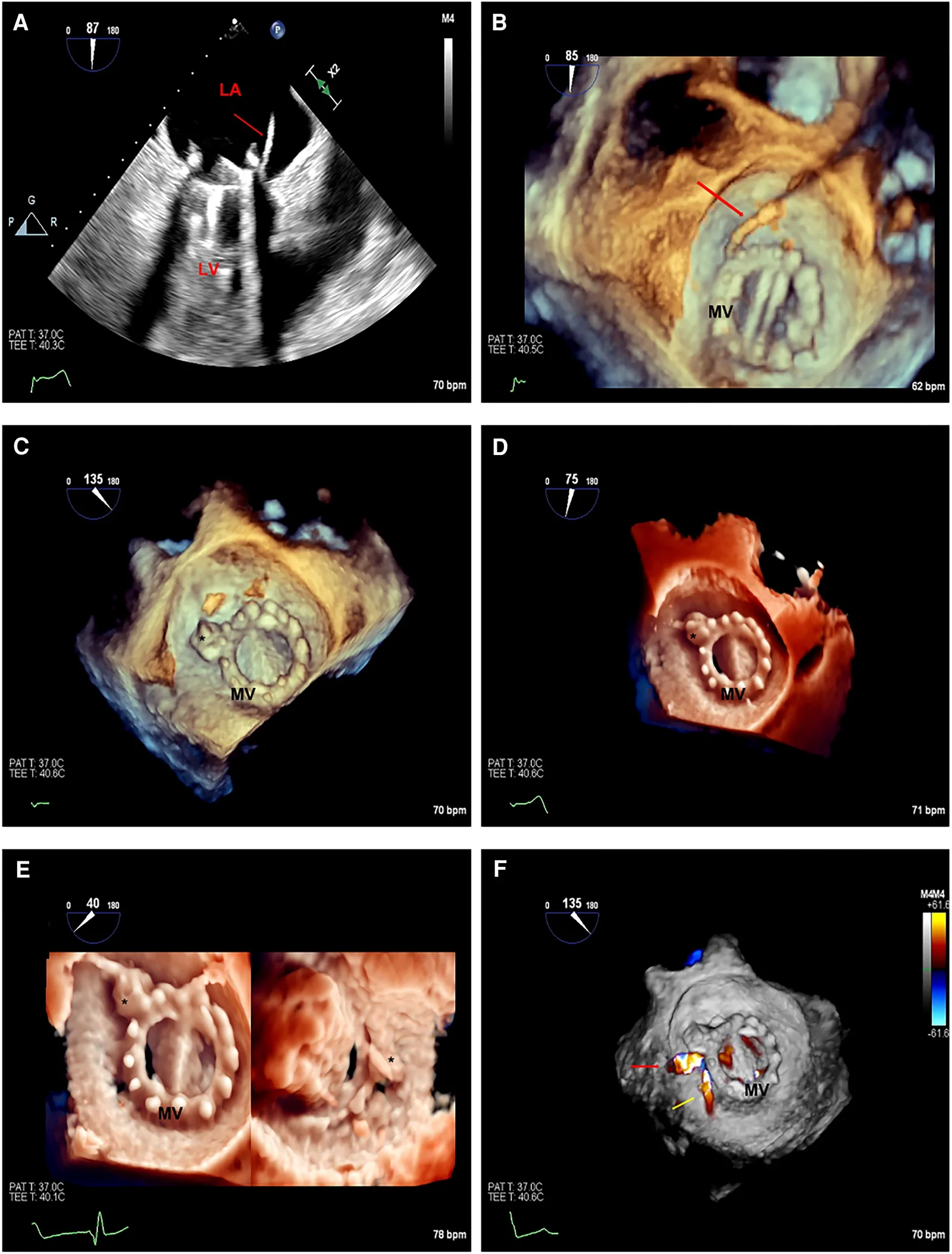
2D (*A*) and 3d-TEE (*B*) images depict a guidewire (red arrow) across the anterolateral paravalvular defect. Real-time 3D-TEE standard (*C*) and transillumination imaging (TI) TrueView (*D*) images show closure of the anterolateral defect using a 10 mm Amplatzer device (asterisk). TrueView images from atrial (left) and ventricular (right) perspectives (*E*) confirm successful closure of the periprosthetic regurgitation (asterisk), while the smaller posterolateral defect remains untreated. The 3D-TEE atrial view (*F*) demonstrates two residual periprosthetic jets: one just posterior to the closure device (red arrow) and the pre-existing posterolateral jet at nine o’clock (yellow arrow).^1^ LA, left atrium; LV, left ventricle; MV, mitral valve.^35^

Pre-procedural evaluation requires exhaustive anatomical mapping using real-time 3D datasets and 3D MPR. Interventional imagers utilize a clock-face notation to describe the exact radial and circumferential location of the defect relative to the sewing ring. Cardiac CT provides complementary insights into leak trajectory and the optimal fluoroscopic plane for device alignment. These measurements are vital for selecting appropriately sized occluder devices and determining the safest access route, such as transseptal or transapical. *Figure 7* shows 2D and 3D TEE imaging of a mechanical mitral prosthesis with anterolateral and posterolateral PVLs, while *Figure 8* demonstrates the wire crossing, closure of the anterolateral defect and the presence of residual periprosthetic jets.

Intraprocedurally, 3D/4D TEE is essential for guiding TSP and ensuring that guidewires correctly traverse the paravalvular gap without damaging adjacent structures. Echo-fluoroscopy fusion imaging has further simplified this process, particularly for radiolucent valves, by co-registering anatomical models with live fluoroscopy to facilitate leak engagement and device positioning.^92–94^

Once the device is deployed, the imager monitors for stable positioning and performs a ‘tug test’. Success is adjudicated using 3D colour Doppler to exclude significant residual shunts and verify that the occluder does not interfere with prosthetic leaflet motion. Continuous monitoring also allows for the immediate detection of rare complications like device embolization or cardiac tamponade. This integrated approach ensures procedural success and patient safety in complex structural repairs.^9,17,90–95^

## Septal reduction therapies: from alcohol ablation to radiofrequency techniques

Hypertrophic cardiomyopathy (HCM) is an autosomal dominant disease characterized by myocyte disarray and myocardial fibrosis. Dynamic outflow obstruction, occurring in up to two-thirds of patients, is primarily caused by basal septal hypertrophy and systolic anterior motion (SAM) of the mitral valve leaflets. While surgical myectomy and alcohol ablation are established treatments, Percutaneous Intramyocardial Septal Radiofrequency Ablation (PIMSRA) has emerged as a novel, minimally invasive alternative.^96,97^

PIMSRA utilizes high-frequency alternating current delivered via a radiofrequency needle to induce irreversible coagulative necrosis within the interventricular septum (IVS), leading to septal reduction and relief of the LVOT obstruction. The success and safety of this procedure are fundamentally dependent on meticulous echocardiographic guidance. Pre-procedural assessment using TTE is mandatory to confirm the degree of hypertrophy and identify the specific pattern of outflow obstruction.^97–99^

During the intervention, 2D and colour Doppler TTE are used to guide the transapical pin is inserted and is advanced within the myocardium to avoid vascular injury, targeting the basal and mid-point parts of the anterior and posterior IVS. To protect the cardiac conduction system, a safety distance of 3–5 mm must be maintained between the ablation zone and the conduction tissue.^100–103^

Post-procedural monitoring focuses on the immediate evaluation of the residual outflow gradient, changes in myocardial thickness and the size of the ablation area. Current data demonstrates that the procedure provides significant symptomatic relief and physiological improvement in cardiac function, accompanied by a tolerable rate of procedural complications. However, the Heart Team must continue to rely on long-term echocardiographic follow-up to ensure durable results, as larger randomized trials are needed to fully validate this technique against traditional surgical standards.^100–103^

## Conclusion

SHDs continue to impose a significant burden, especially in high-risk patients, where surgical options may be limited. The rapid evolution of transcatheter interventions has provided life-altering options for previously ineligible patients. Across all procedures, echocardiography remains the cornerstone of guidance, providing real-time, high-resolution imaging essential for procedural safety and success. As device technologies and imaging modalities advance, the integration of 3D echocardiography, fusion imaging and ICE will further enhance procedural precision. This review emphasizes the growing significance of IE and highlights the need for structured training programs and broader access to advanced imaging modalities. As the field evolves, its impact is poised to grow, offering exciting prospects for both clinicians and patients.

## Data Availability

No new data were generated or analyzed in support of this research.

## Lead author biography

Professor Konstantinos Toutouzas, MD, PhD, FESC, FSCAI is Professor of Cardiology at the National and Kapodistrian University of Athens and Head of the Structural Heart Disease and Cardio-Oncology Unit at the First Department of Cardiology, Hippokration General Hospital of Athens, Greece. He is President of the Hellenic Society of Cardiology and serves as Vice-Chair of the Council of Cardiology Practice of the European Society of Cardiology (2024–2026). He received his MD from the University of Athens, completed his cardiology training and PhD at the First University Cardiology Clinic, and undertook advanced training in interventional cardiology in Milan (Centro Cuore Columbus and San Raffaele). His clinical and research interests focus on intracoronary imaging and vulnerable plaque assessment, transcatheter aortic valve implantation (TAVI) and the broader field of transcatheter structural heart interventions (including ASD/PFO closure, paravalvular leak closure, and leY atrial appendage occlusion). Professor Toutouzas has authored 650+ peer-reviewed publications and contributed to international consensus documents and major textbooks, including chapters for the EAPCI book. He is an experienced peer reviewer and editorial contributor for international cardiovascular journals and a frequent invited speaker and faculty member at leading congresses (including ESC, ACC, TCT and EuroPCR). He also directs postgraduate education in interventional cardiology and supervises doctoral and postgraduate trainees, with a strong track record in training international fellows.
